# Extended‐release buprenorphine in adults with opioid use disorder involving poppy seed tea: An 18‐month retrospective case series

**DOI:** 10.1111/ajad.70187

**Published:** 2026-07-15

**Authors:** John McMaster, Saumiya Seshakumaran, Hesitha Abeysundera, Isha Tipirneni, Rita Hormiz

**Affiliations:** ^1^ Alcohol and Other Drugs Service, Gold Coast Health Gold Coast Queensland Australia; ^2^ Griffith University Gold Coast Queensland Australia; ^3^ Bond University Gold Coast Queensland Australia

## Abstract

**Background and Objectives:**

Poppy seed tea (PST) contains unpredictable opioid concentrations, destabilising tolerance and increasing overdose risk. Extended‐release buprenorphine (BUP‐XR) provides sustained coverage without daily dosing.

**Methods:**

Eighteen‐month follow‐up of adults (*N* = 3) with opioid use disorder (OUD) involving PST in Australia.

**Results:**

All had polysubstance use and transitioned to BUP‐XR after 2 days to 6 months of sublingual buprenorphine. Posttransition urine drug screens were morphine‐negative; one patient was intermittently tramadol‐positive. All remained in treatment at 18 months.

**Discussion and Conclusions:**

BUP‐XR assisted stability within multidisciplinary care.

**Scientific Significance:**

This series adds longer‐term follow‐up on PST‐related OUD and suggests BUP‐XR feasibility.

## INTRODUCTION

Poppy seed tea (PST), brewed from *Papaver somniferum* seeds sold for culinary use, exposes users to unpredictable opioid concentrations, increasing overdose risk and contributing to opioid use disorder (OUD).^1^, ^2^ Despite increasing recognition of OUD presentations involving PST, evidence to guide management remains limited.^3^, ^4^, ^5^, ^6^, ^7^, ^8^, ^9^

PST is prepared from unwashed seeds coated with latex from the seed pod, which contains opium alkaloids including morphine, codeine and thebaine.^1^ Opioid content varies markedly between seed batches and preparation methods, making consistent dosing unreliable.^2^ PST use may also be under‐recognised; in one New Zealand addiction service, half of individuals with OUD reported prior use.^10^

PST may add clinically relevant risks to broader OUD presentations. Unpredictable opioid exposure increases toxicity risk, particularly with concomitant opioids or sedatives.^2^, ^3^ Severe poisonings linked to high‐thebaine seed batches have been reported in Australia,^11^ atypical toxidromes following intravenous use in New Zealand,^12^ and at least 19 PST‐linked deaths have been reported in the United States.^13^

Opioid agonist treatment (OAT) is an evidence‐based treatment for OUD that supports abstinence from illicit or nonprescribed opioids.^14^ Buprenorphine is a partial μ‐opioid receptor agonist with high receptor affinity and a ceiling effect on respiratory depression.^15^ Extended‐release buprenorphine (BUP‐XR) provides sustained coverage across the dosing interval^16^ and reduces reliance on daily dosing,^17^ which may be useful where variable opioid exposure complicates treatment. In Australia, BUP‐XR is available as weekly or monthly Buvidal or monthly Sublocade.^18^

Guidance for OUD presentations involving PST is limited by heterogeneous treatment approaches and sparse longer‐term outcome data.^3^, ^4^, ^5^, ^6^, ^7^, ^8^, ^9^, ^12^ The two largest case series suggest that stabilisation may require buprenorphine doses at the upper end of standard ranges, although neither included BUP‐XR.^3^, ^4^ Where BUP‐XR has been reported, follow‐up has been brief, limiting interpretation of treatment retention and longer‐term clinical course.^8^, ^9^ This series addresses these gaps by describing 18‐month outcomes with BUP‐XR in adults with OUD involving PST.

## METHODS

This case series was conducted at a single‐centre multidisciplinary Australian outpatient addiction service accepting referrals and self‐presentations. Between January and December 2024, patients with OUD involving PST were identified during routine intake assessments and follow‐up medical reviews. PST use was not systematically screened by questionnaire or clinical record review; therefore, additional cases may have been missed.

Patients were eligible if they met all three criteria: diagnosis of OUD according to the Diagnostic and Statistical Manual of Mental Disorders, Fifth Edition^19^; PST clinically identified as a primary opioid associated with withdrawal or functional impairment; and commencement of OAT.

Treatment was managed by experienced clinicians according to state‐wide OUD guidelines.^18^ OAT options included methadone, sublingual buprenorphine (SL‐BUP) and BUP‐XR. Treatment selection, including transition to BUP‐XR, used shared decision‐making. Extended‐release naltrexone was not subsidised in Australia and therefore not routinely offered by the clinic.

Outcome data were extracted retrospectively from medical records in February 2026, allowing 18‐month follow‐up for all included patients. Outcomes of interest included treatment retention, self‐reported substance use, routine urine drug screen (UDS) results and documented psychosocial functioning.

## ETHICS

Human Research Ethics Committee approval was obtained, and all patients provided written consent for publication.

## CASE PRESENTATION

Three eligible patients were identified, and all were included. Clinical characteristics, treatment and outcomes are summarised in Table 1.

**Table 1:** Cross‐case summary of clinical characteristics, treatment parameters and outcomes.

Parameter	Patient 1	Patient 2	Patient 3
Baseline characteristics			
Sex	Male	Female	Male
Age	30s	30s	20s
Country of birth	Australia	Australia	Australia
Aboriginal and/or Torres Strait Islander	No	No	No
Duration of PST use	~10 years	~6 months	~6 months
Estimated daily seed quantity	~2.5 kg	~1.0 kg	~1.5 kg
Psychiatric comorbidity	Schizophrenia	Complex PTSD; depression; anxiety	Anxiety; depression
Other reported substance use	Heroin (IV); pregabalin; benzodiazepines; methamphetamine; cannabis; alcohol	Heroin (rectal administration); tapentadol; oxycodone; cannabis	Heroin (IV); fentanyl patches; tapentadol; kratom; coca leaves; cannabis
Induction UDS	Benzodiazepines+; cannabis+; morphine+; pregabalin+	Benzodiazepines+; cannabis+; morphine+	Immunoassay: benzodiazepines+; cannabis+; morphine+. MS supplement^a^: morphine‐3‐glucuronide+; hydrocodone+; fentanyl/norfentanyl+; thebaine+; pregabalin+; cannabinoids+
Treatment course			
Initial SL‐buprenorphine dose	8 mg/day	24 mg/day	24 mg/day
Re‐induction required	No	No	Yes—following disengagement
Time to BUP‐XR	2 days	6 months	3 months from initial induction; 5 days following re‐induction
BUP‐XR formulation^b^	Buvidal: weekly → monthly	Sublocade: monthly	Buvidal: weekly → monthly
BUP‐XR maintenance dose	128 mg monthly	2 × 300 mg monthly loading doses → 100 mg monthly	128 mg monthly
BUP‐XR adherence/continuity	No sustained disengagement documented after transition; remained on monthly BUP‐XR at 18 months	No sustained disengagement documented; remained on monthly BUP‐XR at 18 months	Transferred to private OAT provider at 10 months; ongoing monthly BUP‐XR confirmed at 18 months
Clinical outcomes			
Retention in OAT at 18 months	Yes	Yes	Yes
Opioid use at follow‐up	Reported PST cessation. UDS (*n* = 5): tramadol immunoassay positive (*n* = 3)^c^	Reported opioid abstinence. UDS (*n* = 7): negative	Early relapse to prescribed opioids. Post‐BUP‐XR: reported abstinence. UDS (*n* = 3): negative
Other substance use, self‐report/UDS	Cannabis only. UDS (*n* = 5): cannabis+; benzodiazepines+ (*n* = 2)	Cannabis only. UDS (*n* = 5): cannabis+; pregabalin+ (*n* = 2)	Cannabis only. UDS (*n* = 1): cannabis+
Psychosocial outcomes	No further overdoses; no psychiatric admissions; discharged from CMHS case management; stable accommodation	No psychiatric admissions; stable accommodation; commenced trauma‐focused psychotherapy	Active job seeking; sustained stability at transfer to private care

Abbreviations: BUP‐XR, extended‐release buprenorphine; CMHS, community mental health services; IV, intravenous; MS, mass spectrometry; OAT, opioid agonist treatment; PST, poppy seed tea; PTSD, posttraumatic stress disorder; SL, sublingual; UDS, urine drug screen.

^a^ Patient 3 induction UDS was supplemented with mass spectrometry; all other induction UDS results were assessed by immunoassay only.

^b^ Buvidal (Camurus, Sweden) is administered as a weekly or monthly subcutaneous injection; Sublocade (Indivior, USA) is administered as a monthly subcutaneous injection.

^c^ Tramadol immunoassay results were not confirmed by mass spectrometry; source was not established from available records.

## PATIENT 1

A man in his 30 s with schizophrenia, managed long‐term by community mental health services (CMHS), with four recent psychiatric admissions, was referred for addiction consultation. He reported a 10‐year history of daily PST use, alongside sporadic intravenous heroin and polysubstance use. He described escalating to heroin after PST no longer produced the desired euphoric effects.

He initially declined OAT. During a subsequent psychiatric admission, he experienced an unintentional opioid overdose after heroin use on approved leave and required intensive care admission. While hospitalised, he received SL‐BUP 8 mg for 2 days as a brief inpatient lead‐in before transition to weekly BUP‐XR 16 mg and subsequent stabilisation on monthly BUP‐XR 128 mg. At 6 months, he reported cessation of PST use and reduced overall substance use. Serial UDS (*n* = 5) were intermittently tramadol‐positive by immunoassay (*n* = 3), which he denied using, and otherwise negative for opioids. At 18 months, he remained engaged in treatment, had stable accommodation, had no further overdoses or psychiatric admissions, and had been discharged from CMHS case management.

## PATIENT 2

A woman in her 30s was referred from CMHS for addiction consultation. She reported 6 months of daily PST use, multiple unsuccessful unassisted cessation attempts and withdrawal severity as the main barrier to stopping. She described perceived legality, affordability and accessibility as key drivers of use. She also intermittently used heroin, tapentadol, oxycodone and other substances and had two psychiatric admissions for suicidality in the preceding 12 months.

She stabilised on SL‐BUP 24 mg within 6 days and initially declined BUP‐XR because of needle anxiety. Over subsequent months, she reported no other opioid use, minimal cravings and improved psychosocial functioning. At 6 months, after her partner relapsed, she became increasingly concerned about her own relapse risk and requested transition to BUP‐XR. She received two 300 mg monthly loading doses followed by 100 mg monthly maintenance. At 18 months, she remained on monthly BUP‐XR 100 mg, was in stable accommodation, had commenced trauma‐focused psychotherapy and reported reduced overall substance use. Serial UDS (*n* = 7) were negative for other opioids. She described transition to BUP‐XR as central to her recovery, citing sustained buprenorphine coverage as protective against relapse‐related anxiety.

## PATIENT 3

A man in his 20s self‐presented requesting OAT. He reported a 6‐month history of daily PST use with intermittent intravenous heroin, fentanyl patch and tapentadol use. He had comorbid depression and anxiety, and a family history of OUD. He reported discovering PST through an online retailer while purchasing psychoactive substances including kratom and coca leaves.

He stabilised on SL‐BUP 24 mg over 3 weeks. After 2 months, he disengaged from treatment and relapsed following prescription of opioid analgesics for an orthopaedic fracture. One month later, he re‐presented and commenced weekly BUP‐XR 32 mg, later transitioning to monthly BUP‐XR 128 mg maintenance. At 6 months, he reported no other opioid use, minimal cravings, stable accommodation and active job seeking. UDS obtained after transition to BUP‐XR (*n* = 3) were negative for other opioids. At 10 months, he transferred to a private OAT provider and professional correspondence confirmed continuation of monthly BUP‐XR 128 mg at 18 months.

## DISCUSSION

This case series extends the limited literature on OUD involving PST^3^, ^4^, ^5^, ^6^, ^7^, ^8^, ^9^, ^12^ by providing 18‐month follow‐up after BUP‐XR treatment in adults with psychiatric comorbidity and polysubstance use. Consistent with prior series,^3^, ^4^ Patients 2 and 3 required upper‐range buprenorphine doses, whereas Patient 1 received only a brief 8 mg inpatient lead‐in before rapid BUP‐XR transition after overdose. At 18 months, all remained in OAT, reported cessation of PST use and had documented clinical stability.

Concurrent opioid and polysubstance use complicated interpretation, as tolerance and withdrawal could not be attributed to PST alone. However, this complexity is clinically important. PST is associated with unpredictable opioid exposure, unreliable self‐titration and destabilised tolerance. These risks may be heightened by concomitant opioids or sedatives, relapse or interruptions in use. One patient experienced an unintentional heroin overdose after interruption of PST use before induction, possibly reflecting destabilised opioid tolerance.

In this context, BUP‐XR may offer practical advantages through sustained buprenorphine exposure, high‐affinity receptor occupancy and reduced reliance on daily adherence.^15^, ^16^ In this series, it was incorporated at different treatment points, illustrating flexibility rather than a uniform approach. One patient described BUP‐XR as central to her recovery, citing sustained coverage as protective against relapse‐related anxiety.

Toxicological interpretation was limited because routine UDS could not reliably distinguish PST from other opioid sources. Patient 3's induction mass spectrometry detected thebaine, supporting recent PST exposure. After BUP‐XR transition, Patients 2 and 3 had no UDS evidence of other opioid use in available testing. One patient maintained reported PST cessation, supported by morphine‐negative urine drug screens; however, intermittent tramadol‐positive immunoassays limited confirmation of complete opioid abstinence. All patients received case‐management support, and Patient 1 also received community psychiatric care; therefore, findings cannot be attributed to BUP‐XR alone.

## LIMITATIONS

Interpretation was limited by the small sample size and absence of a comparator group. PST use was not systematically screened and prevalence cannot be inferred. PST exposure and estimated quantity relied on self‐report and were not routinely confirmed or quantified toxicologically. Outcome assessment was retrospective and based on clinical documentation, self‐report and nonprotocolised UDS. UDS was not PST‐specific or routinely confirmed by mass spectrometry; incidental results, including tramadol positivity in one patient, should therefore be interpreted cautiously. BUP‐XR adherence and continuity were assessed from routine records and correspondence. Direct BUP‐XR induction was not examined, and findings from a multidisciplinary specialist service may not generalise to less resourced settings.

## CONCLUSION

PST may be a clinically important and unpredictable opioid exposure within complex OUD presentations, particularly where polysubstance use is present. In this small case series, BUP‐XR was feasible within routine multidisciplinary OUD care and may be practically useful where variable opioid exposure, relapse risk or daily‐dosing barriers complicate treatment. Further descriptive and comparative studies are needed to clarify outcomes and optimal treatment strategies for OUD presentations involving PST.

## CONFLICT OF INTEREST STATEMENT

The authors declare no conflicts of interest.

## Data Availability

The data that support the findings of this study are available from the corresponding author upon reasonable request.
